# Paralogous *SQUAMOSA PROMOTER BINDING PROTEIN*-*LIKE* (*SPL*) genes differentially regulate leaf initiation and reproductive phase change in petunia

**DOI:** 10.1007/s00425-015-2413-2

**Published:** 2015-10-07

**Authors:** Jill C. Preston, Stacy A. Jorgensen, Rebecca Orozco, Lena C. Hileman

**Affiliations:** Department of Plant Biology, The University of Vermont, 111 Jeffords Hall, 63 Carrigan Drive, Burlington, VT 05405 USA; Ecology and Evolutionary Biology, The University of Kansas, 8009 Haworth Hall, 1200 Sunnyside Avenue, Lawrence, KS 66045 USA

**Keywords:** Flowering, Paralogs, Petunia, Plastochron, Virus-induced gene silencing (VIGS)

## Abstract

**Duplicated petunia clade-VI*SPL*genes differentially promote the timing of inflorescence and flower development, and leaf initiation rate.**

The timing of plant reproduction relative to favorable environmental conditions is a critical component of plant fitness, and is often associated with variation in plant architecture and habit. Recent studies have shown that overexpression of the microRNA *miR156* in distantly related annual species results in plants with perennial characteristics, including late flowering, weak apical dominance, and abundant leaf production. These phenotypes are largely mediated through the negative regulation of a subset of genes belonging to the *SQUAMOSA PROMOTER BINDING PROTEIN*-*LIKE* (*SPL*) family of transcription factors. In order to determine how and to what extent paralogous *SPL* genes have partitioned their roles in plant growth and development, we functionally characterized petunia clade-VI *SPL* genes under different environmental conditions. Our results demonstrate that *PhSBP1*and *PhSBP2* differentially promote discrete stages of the reproductive transition, and that *PhSBP1*, and possibly *PhCNR*, accelerates leaf initiation rate. In contrast to the closest homologs in annual *Arabidopsis thaliana* and *Mimulus guttatus*, *PhSBP1* and *PhSBP2* transcription is not mediated by the gibberellic acid pathway, but is positively correlated with photoperiod and developmental age. The developmental functions of clade-VI *SPL* genes have, thus, evolved following both gene duplication and speciation within the core eudicots, likely through differential regulation and incomplete sub-functionalization.

**Electronic supplementary material:**

The online version of this article (doi:10.1007/s00425-015-2413-2) contains supplementary material, which is available to authorized users.

## Introduction

Variation in plant form results largely from the differential timing of developmental phase transitions that can occur gradually (e.g., leaf size) or rapidly (e.g., flowering) in response to both endogenous and exogenous signals (Poethig 2003; Bäurle and Dean 2006). The ability of plants to match the timing of these transitions to favorable environmental conditions is a critical component of fitness, and is often associated with life history and architectural trait differences within and between populations (Hall and Willis 2006; Franks et al. 2007; Forrest and Miller-Rushing 2010). In some cases, genes involved in growth and differentiation are tightly synchronized, resulting in a predictable number of organs at a given stage of shoot growth. In contrast, some genes involved in differentiation are unaffected by shoot growth. An example of the latter are many genes involved in flowering time that have no effect on the rate of leaf initiation. Mutations in these genes result in early- or late flowering, and cause a concomitant decrease or increase of leaves, respectively (Koorneef et al. 1991; Haselhorst et al. 2011). Understanding to what extent genes involved in phase change can be uncoupled from genes involved in shoot growth under different environmental conditions is a key question in plant developmental biology (Poethig 2003).

Several genetic pathways that converge on floral integrator genes involved in floral competency and meristem identity tightly control phase change in angiosperms. In the annual rosid Arabidopsis (*Arabidopsis thaliana*, Brassicaceae), the recently duplicated clade-VI *SQUAMOSA*-*PROMOTER BINDING PROTEIN (SBP)*-LIKE (*SPL*) genes—*AtSPL3, AtSPL4*, and *AtSPL5*—integrate signals from the age, autonomous, photoperiod, and gibberellic acid signal transduction pathways to redundantly promote the formation of flowers within the inflorescence (Wu and Poethig 2006; Gandikota et al. 2007; Wang et al. 2009; Yamaguchi et al. 2009, 2014; Jung et al. 2012; Porri et al. 2012; Yu et al. 2012). Under short-day conditions, all three *SPL* genes are negatively regulated in an age-dependent manner by the microRNA *miR156*, and are positively regulated by SUPPRESSION OF OVEREXPRESSION OF CONSTANS 1 (SOC1) (Wu and Poethig 2006; Gandikota et al. 2007; Wang et al. 2009; Yamaguchi et al. 2009; Jung et al. 2012). Conversely, under long-day conditions, SOC1, FLOWERING LOCUS T (FT), and FLOWERING LOCUS D (FD) positively regulate *SPL* genes in leaves (Jung et al. 2012). In a positive feedback loop, SPL proteins then indirectly activate leaf *FT* expression, possibly through the direct binding of the inflorescence meristem gene *FRUITFULL* (*FUL*), and directly activate transcription of *FUL*, *SOC1*, *APETALA1* (*AP1*) and *LEAFY* (*LFY*) in the shoot apex to promote flower production (Corbesier and Coupland 2006; Corbesier et al. 2007; Wang et al. 2009; Yamaguchi et al. 2009).

Although *atspl3* mutants have no aberrant phenotypes, overexpression of *AtSPL3* lacking the *miR156*-binding site accelerates juvenile to adult phase change, and results in precocious flowering without affecting the rate of leaf development (Wu and Poethig 2006; Gandikota et al. 2007; Schwarz et al. 2008; Wang et al. 2008, 2009; Yamaguchi et al. 2009). Precocious flowering is also evident in *AtSPL4* and *AtSPL5* overexpression lines (Wu and Poethig 2006). However, with the exception of abaxial leaf trichomes, overexpression of *AtSPL4* and *AtSPL5* does not decrease the number of leaves with juvenile characteristics (Wu and Poethig 2006). In accordance with the overexpression results, *miR156*-regulated silencing of multiple *SPL* genes (including *AtSPL3/4/5*) delays phase transitioning, but maintains apical dominance (Wu and Poethig 2006). Together these data suggest that Arabidopsis clade-VI *SPL* genes function redundantly in promoting reproductive, and possibly vegetative, phase change without affecting leaf or branch number. However, because of functional redundancy, further evidence is needed to determine the exact role of each gene in these developmental transitions.

Evidence from the perennial asterid species snapdragon (*Antirrhinum majus*, Plantaginaceae) and tomato (*Solanum lycopersicum*, Solanaceae) support conservation and diversification of core eudicot clade-VI *SPL* gene function following speciation (Klein et al. 1996; Manning et al. 2006; Preston and Hileman 2010). Similar to Arabidopsis, silencing of the *AtSPL3/4/5* snapdragon homolog *AmSBP1* has a negative effect on flowering time (Preston and Hileman 2010). Although inflorescence development is not delayed, flower production is completely abolished in *AmSBP1*-silenced plants. Furthermore, *AmSBP1*-silenced plants display abnormal vegetative phenotypes due to the loss of apical dominance, developing lateral vegetative branches after the main axis bearing an inflorescence fails to flower (Preston and Hileman 2010). Conversely, in tomato, epigenetic mutations in the *AtSPL3/4/5* homolog *LeSPL*-*COLORLESS NON*-*RIPENING* (*LeSPL*-*CNR*) result in failed fruit ripening (Manning et al. 2006; Chen et al. 2015). Despite these functional insights, it is unclear how *AmSBP1* and *LeSPL*-*CNR* are regulated during development compared to *AtSPL3/4/5*, and whether the clade-VI *SPL* paralogs of *AmSBP1* and *LeSPL*-*CNR* also affect flowering time, branching, and fruit development.

In order to better understand the extent to which clade-VI *SPL* genes have functionally diversified following both gene duplication and speciation, and the underlying mechanism for these functional changes, we characterized the expression and function of two clade-VI *SPL* genes from the perennial asterid petunia (*Petunia* × *hybrida*, Solanaceae) under different growth conditions. Unlike Arabidopsis and snapdragon, which grow from a single dominant stem and have racemose inflorescences, petunia grows from multiple stems and has cymose inflorescences (Castel et al. 2010; Preston and Hileman 2010). Combining data from Arabidopsis, snapdragon, tomato, and petunia thus allows comparison of clade-VI *SBP*-box gene function across both phylogenetically and morphologically diverse species. Our results demonstrate that the petunia clade-VI *SPL* genes, *PhSBP1*, *PhSBP2*, and possibly *PhCNR*, have overlapping but divergent functions in the reproductive transition and plastochron length (leaf initiation rate). Furthermore, we show that gibberellic acid regulation of clade-VI *SPL* genes differs across annual and perennial species of core eudicots.

## Materials and methods

### Plant material and growth conditions

*Petunia* × *hybrida* (petunia) ‘Fantasy blue’ (2PET131), *Mimulus guttatus* IM767, and *Antirrhinum majus* (snapdragon) ANT11 seed was initially obtained from Seedman.com, J.K. Kelly at the University of Kansas, and the Gatersleben collection in Germany, respectively. For the photoperiod experiments, 20 wild type plants were grown under continuous light, long days (16 h light/8 h dark) or short days (8 h dark/16 h light) at 21–22 °C in a growth chamber until death or flowering with two experimental replicates. For the gibberellic acid experiments, 6-month-old non-flowering short-day grown plants were separated into two treatments. Plants in treatment one were sprayed twice weekly with 20 uM GA_3_, whereas plants in treatment two were sprayed twice weekly with water. Ice plant (Aizoaceae, Caryophyllales), carnation (Caryophyllaceae, Caryophyllales), *Ruellia trittoniana* (ruellia, Acanthaceae, Lamiales)*, Plumeria rubra* (frangipani, Apocynaceae, Gentianales)*, Bidens torta* (beggartick, Asteraceae, Asterales)*, Primula hortensis* (Primulaceae, Ericales), and *Penstemonbarbatus* (bear-tongue, Plantaginaceae, Lamiales) were grown under standard greenhouse conditions at the University of Kansas.

### Gene isolation and phylogenetic analysis

In order to isolate all homologs of clade-VI *SPL* genes from petunia and other representative asterids, multiple degenerate primers were designed based on previously published and aligned *SPL* gene sequences from core eudicots (Supplemental Table S1). Total RNA was extracted from flower buds and leaves using TriReagent (Life Technologies), and contaminating DNA was removed with DNase I (Qiagen). One μg of RNA was used as a template for iScript cDNA synthesis (BioRad), the resulting cDNA was diluted 1:10, and 2 μl was used in PCR reactions. Amplicons derived from standard PCR reactions using different combinations of degenerate primers were cloned into pGEM-T (Promega), and 10–20 colonies per cloning reaction were sequenced. *SPL* genes were identified using BLAST searches and aligned with related genes from asterids and rosids in MacClade (Maddison and Maddison 2003). Phylogenetic relationships between *SPL* genes of petunia and other species were estimated using maximum likelihood methods in GARLI (Zwickl 2006). Analyses were run using the best-fitting model of molecular evolution (GTR + I + Γ), according to results of ModelTest 3.7 (Posada and Crandall 1998). The maximum likelihood analysis was run with 10 random additions, and bootstrap values were obtained using 500 bootstrap replicates. Isolation of putative petunia SPL target genes was accomplished using gene-specific primers designed from previously published sequences, and degenerate primers designed from aligned core eudicot *AP1/FUL*-like genes (Supplemental Table S2). Newly generated sequences longer than 200 bp in length were deposited in Genbank under the accession numbers KT717959–KT717966; sequences shorter than 200 bp in length can be found in Supplemental Table S3.

### TRV2 plasmid construction

In order to control for any potentially adverse treatment effects of virus-induced gene silencing (VIGS), 194 bps of the petal pigment gene *CHALCONE SYNTHASE* (*CHS)* was PCR amplified from petunia floral cDNA, sequence verified, and cloned into the TRV2 vector as previously described (Chen et al. 2004). For *SPL* gene silencing, four constructs were made: *PhSBP1code*-TRV2 and *PhSBP2code*-TRV2, containing a 250 bp fragment of *PhSBP1* or *PhSBP2* just downstream of the SBP-box domain, and *PhSBP1utr*-TRV2 and *PhSBP2utr*-TRV2 containing a 200 bp fragment of the *PhSBP1* or *PhSBP2* 3′-UTR. Target regions of the *SPL* genes were PCR amplified from petunia (Supplemental Table S1) and cloned into TRV2 using the restriction enzymes *Bam*HI and *Xho*I. Each construct was sequence verified and transformed into *Agrobacterium tumefaciens* strain EHA105.

### VIGS and phenotyping

*Agrobacterium* growth and plant infiltration methods followed Hileman et al. (2005) and Drea et al. (2007). Batches of 25 plants at the 4–6 leaf stage were infiltrated in half their leaves with a 1:1 ratio of TRV1:TRV2 using a needleless syringe, with at least three experimental replicates for each construct conducted at different times of the year. Following infiltration, plants were grown under long-day or short-day conditions for 2 weeks at which time RNA was extracted from the youngest (upper) leaf to screen for infection with TRV1 and TRV2 using the primers OYL195F and OYL198R for TRV1, and pYL156F and pYL156R for TRV2 (Hileman et al. 2005). Flowering time for each plant was determined as the production of the first visible floral bud, leaf and vegetative branch number at flowering were scored when the first flower was fully open, and days to transition was determined following emergence of the first inflorescence bracts. Leaf area was measured as the ratio of laminar width to laminar length. Since all dependent variables had skewed distributions, data was log-transformed prior to analysis. ANOVA was used to test for significant differences in days to the inflorescence transition, days to flowering, leaf number, branch number, and leaf ratio between treatments. When differences were significant (*P* < 0.05) comparisons were carried out between the *PhCHS*-VIGS control and each *SPL* treatment using a one-tailed Dunnett test in the multcomp package of R version 2.13.0 (R Development Core Team 2011). In cases where mean leaf number was opposite to predictions, i.e., lower in the *SPL* treatment relative to the control treatment, significance differences were determined using a two-tailed Dunnett test.

### RNA extraction and quantitative real-time PCR

Expression analyses were carried out on wild type and VIGS plants under continuous light, 16 h long days, 8 h short days, and short days plus gibberellic acid conditions using quantitative reverse transcriptase (qRT)-PCR on a StepOne Plus machine (Life Technologies). Total RNA was extracted from leaves, shoot apical meristems, and dissected flowers at different developmental stages, and used to make cDNA as previously described. To determine times during the diurnal cycle when *PhSBP1* and *PhSBP2* expression would be high enough to compare expression levels across treatments, RNA was collected from fully expanded mid stage (fourth leaf from base) and upper leaves of two independent plants at the eight leaf stage every 4–5 h during the light period. All other material was collected between 9 and 10 am, which corresponds to 3–4 h after the zeitgeber in long-day and short-day grown plants. Upper leaf samples were taken when leaves were approximately 1 cm long.

Primer pairs for qRT-PCR were designed in Primer3 (Rozen and Skaletsky 2000) and tested for PCR efficiency using Fast SYBR Green Master Mix (Life Technologies) as previously described (Preston and Hileman 2010) (Supplemental Table S2). The housekeeping genes *EF1alpha* and *UBQ5* were selected as internal controls based on high PCR efficiency and low transcriptional variation across different tissues in petunia (Mallona et al. 2010) and previous studies in *M. guttatus* and snapdragon (Preston and Hileman 2010; Scoville et al. 2011). After correcting for PCR efficiency, cycle threshold (cT) values in target tissues were normalized against the geomean of housekeeping gene expression (Scoville et al. 2011), and the mean was calculated for three to four technical replicates. For VIGS experiments, fold change was calculated in the youngest 1 cm long leaf or vegetative shoot apices by dividing the normalized values of the infected plants with that of control plants at the same developmental stage based on leaf number. For wild type gene expression experiments, biological replicates comprised similar sized tissues from different individuals at the same developmental stage or age as indicated.

## Results

### Isolation and phylogenetic analysis of core eudicot *SPL* genes and their putative targets

**Fig. 1:**
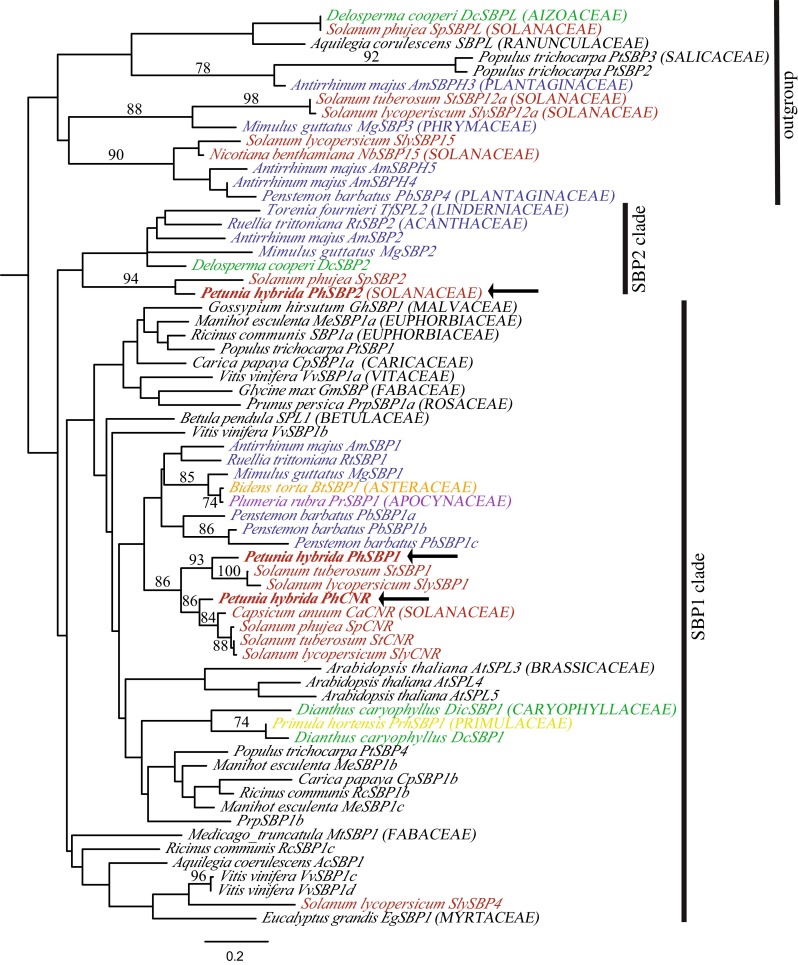
Maximum likelihood (ML) phylogeny of core eudicot clade-VI *SPL* genes. Genes fall into two major lineages labeled SBP1 and SBP2. The SBP1 lineage contains genes from both representative rosids (*black labels*) and asterids (*colored labels*), whereas the SBP2 lineage only contains genes from asterids. ML bootstrap values above 50 % are shown. *Yellow* Ericales; *green* Caryophyllales; *red* Solanales; *blue* Lamiales; *purple* Gentianales; *orange* Asterales. Petunia genes are in *bold red* with *arrows*. Outgroups are indicated

In order to identity all clade-VI *SPL* genes in petunia, and to verify their orthology relative to previously characterized genes, we cloned, sequenced, and phylogenetically analyzed *SPL* genes from multiple species of core eudicot. Sequencing of amplicons from both flower- and leaf-derived cDNA using multiple degenerate primer pairs identified three clade-VI *SPL* genes in petunia (Fig. 1), two of which (*PhSBP1* and *PhSBP2*) were chosen to be the main focus of this study. Nucleotide comparison revealed that *PhSBP1* shares 63 % identity with *PhSBP2*, and 83 % identity with *PhCNR*. Phylogenetic analysis based on the SBP-box domain of multiple core eudicot genes support orthology between petunia *PhSBP1* and *Solanum lycopersicumSlySBP3* (93 % ML bootstrap), *PhCNR* and *SlyCNR* (86 % ML bootstrap), and *PhSBP2* and *Solanum phujea SpSBP2* (94 % ML bootstrap) (Fig. 1). Although not well supported by ML bootstrapping, copy number and the most likely topology suggest at least two major lineages of *SPL* genes (hereafter the SBP1 and SBP2 clades), similar to that reported by Preston and Hileman (2010) (Fig. 1).

With the exception of tomato *SlySBP4*, crown group asterid genes in the SBP1 clade, including *PhSBP1, PhCNR* and *AmSBP1*, largely track the species phylogeny (Fig. 1). However, relationships among the crown asterid, early-diverging asterid *Dianthus caryophyllus*, *Delosperma cooperi*, and *Primula hortensis*, and rosid SBP1 clade genes are generally not well supported. The remaining asterid clade-VI *SPL* genes fall successively sister to the SBP1 lineage with little support among them (Fig. 1). Based on gene copy number, tree topology, and branch support, we infer that the two clade-VI *SPL* genes are derived from a duplication that predates diversification of the core eudicots. If the topology of the most likely tree is correct then we infer the loss of SBP2 clade genes, which include *PhSBP2* and *AmSBP2*, from rosid eudicots. Alternatively, rosids genes forming a basal grade within the SBP1 clade, such as *Medicago truncatula MtSBP1*, are orthologous to asterid SBP2 clade genes (Fig. 1).

Sequencing of multiple clones from both flower- and leaf-derived petunia cDNA using gene-specific primers revealed one copy of previously uncharacterized *FT*, and one copy of *ALF, FBP26, FBP29*, *PFG*, *UNSHAVEN*, *FBP21*-*SOC1*, and *FBP28*-*SOC1* as previously described (Gerats et al. 1988; Souer et al. 1998; Immink et al. 1999, 2003; Ferrándiz et al. 2000; Litt and Irish 2003; Vandenbussch et al. 2003). However, despite multiple attempts, both specific and degenerate primers failed to amplify *PhFUL* and *PhFL* (Litt and Irish 2003), and no *SQUA* orthologs containing the *euAP1* transcriptional activation or farnesylation motif were isolated. Although the absence of other *AP1/FUL*-like genes from our search does not discount their presence in the genome, these results strongly suggest that *FBP26*, *FBP29* and *PFG* are the only *AP1/FUL*-like genes expressed in leaf and floral tissues, concomitant with relatively high levels of *PhSBP1* and *PhSBP2* transcripts (Supplemental Fig. S1).

### *PhSBP1* and *PhSBP2* expression in wild type petunia

**Fig. 2:**
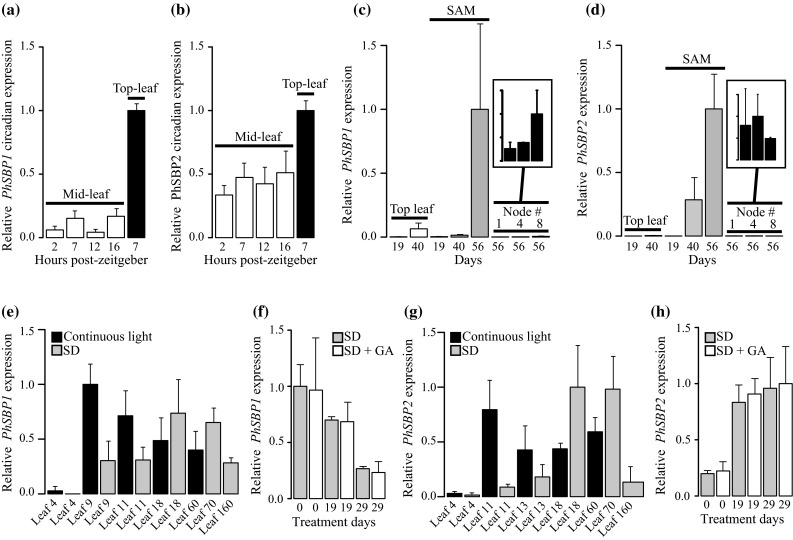
Relative *PhSBP1* and *PhSBP2* expression in wild type tissues. Leaf *PhSBP1* (**a**) and *PhSBP2* (**b**) expression levels are similar across 16 h long days, but increase from the middle (4th leaf) to the apex (8th leaf) of individual plants. *n* = 2 for *bars* and SDs. *PhSBP1* (**c**) and *PhSBP2* (**d**) expression increases with developmental age in leaves and shoot apical meristems (SAM), but only *PhSBP1* transcripts become more abundant in axillary meristems (associated with numbered leaf nodes) from the base to the apex of individual plants. *n* = 4 for *bars* and SDs. **e** *PhSBP1* is more abundant in early development with continuous versus short-day (SD) photoperiods. *n* = 4 for *bars* and SDs. **f** *PhSBP1* expression does not response to gibberellic acid treatment under short days. *n* = 4 for *bars* and SDs. **g** *PhSBP2* is also more abundant in early development with continuous versus short-day photoperiods. **h** *PhSBP2* expression also does not response to gibberellic acid treatment under short days. *n* = 4 for *bars* and SDs

In Arabidopsis and snapdragon, expression of SBP1 clade transcripts increase over developmental time, consistent with a role for these genes in collectively promoting the transition from juvenile to adult, vegetative to reproductive, and/or bract to flower growth (Wu and Poethig 2006; Gandikota et al. 2007; Schwarz et al. 2008; Wang et al. 2008, 2009; Yamaguchi et al. 2009; Preston and Hileman 2010). To determine if petunia *PhSBP1* and *PhSBP2* transcripts similarly increase during development, we conducted qRT-PCR analyses on different wild type tissues separated in time (leaves and shoot apices) and space (leaves and nodes) (Fig. 2a–d). Since expression was detectable, but did not significantly differ across 16 h long days (Fig. 2a, b), all experimental tissues were harvested at a fixed time, 3–4 h post-zeitgeber. In support of our developmental predictions, expression of *PhSBP1* and *PhSBP2* in plants grown under 16 h long days increased at least twofold in fully expanded upper leaves and shoot apices from day 19 to 40 (leaves) or 56 (apices) post-germination (Fig. 2c, d). Furthermore, expression of both genes was higher in late versus early emerging leaves on the same plant (Fig. 2a, b), and in older emerging nodes for *PhSBP1* (box in Fig. 2c). The only expression pattern that failed to match our developmental prediction was *PhSBP2* in nodes one (earliest) to eight (latest), with expression being similar across these tissues (box in Fig. 2d).

To test the hypothesis that photoperiod and gibberellic acid affect expression of *PhSBP1* and *PhSBP2*, we assayed gene expression in plants grown under continuous light versus 8 h short days, and under short days with and without the addition of gibberellic acid. As expected, expression of both genes was higher under continuous relative to short-day light at early stages of development (leaf 4 to 11) (Fig. 2e, g). However, by leaf stage 18, expression was slightly higher in short-day versus continuous light grown plants, despite the fact that short-day plants failed to flower after 200 days. Furthermore, although it stimulated flowering in short-day plants (see next section), exogenous gibberellic acid addition had no effect on *PhSBP1* or *PhSBP2* expression (Fig. 2f, h). To determine if this lack of gibberellic acid response is common across asterids, similar experiments were conducted on perennial snapdragon and annual *M. guttatus*. Gibberellic acid had no effect on snapdragon *AmSBP1* and *AmSBP2*, or *M. guttatus MgSBP2*, but expression of *MgSBP1* increased over twofold relative to mock treated plants without stimulating flowering (Supplemental Fig. S2).

### Petunia clade-VI *SPL* genes differentially control the timing of developmental phase change and leaf initiation

**Fig. 3:**
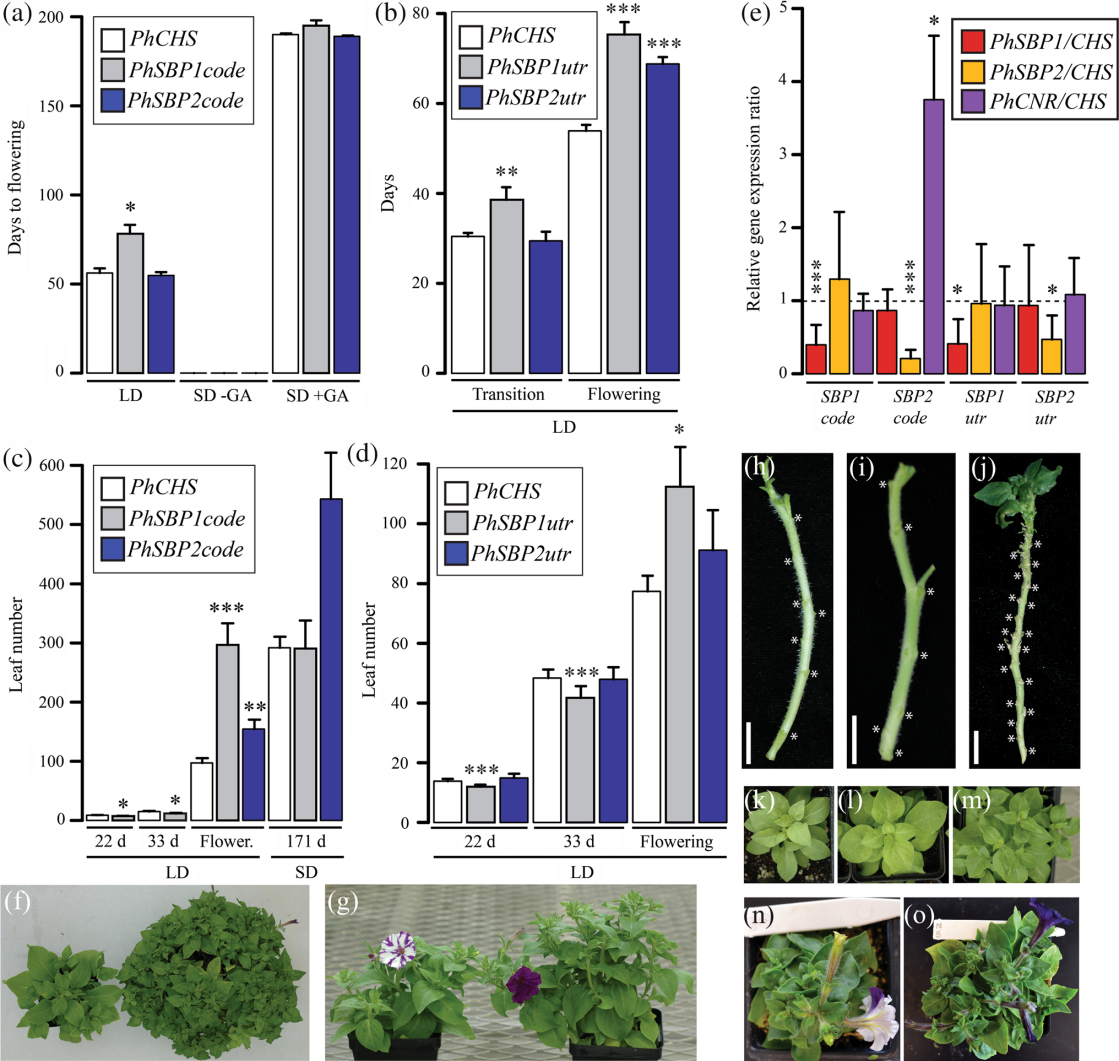
*PhSBP1* and *PhSBP2* VIGS phenotypes. **a** Days to flowering increase in *PhSBP1code*- relative to *PhCHS*- and *PhSBP2code*-silenced individuals under 16 h long days (LD) only (*n* = 29–53 for *bars* and SDs). Infected plants grown under 8 h short days (SD-GA) fail to flower even after 200 days unless treated with gibberellic acid (SD + GA) (*n* = 10 for *bars* and SDs). **b** *PhSBP1utr*-silenced plants take longer to transition to inflorescence development (Transition) in long days relative to *PhCHS*- and *PhSBP2utr*-silenced individuals, whereas both *PhSBP1utr*- and *PhSBP2utr*-silenced plants take longer to flower (Flowering) relative to *PhCHS*-infected controls. *n* = 45–52 for *bars* and SDs. **c** Leaves are initiated more quickly in *PhSBP1code*-silenced plants relative to *PhCHS*-silenced controls based on leaf counts at 22 and 33 days post-germination under long days. Increased leaf number in flowering (Flower.) *PhSBP1code*-infected plants correlate with their late-flowering phenotype. However, since *PhSBP2code*-silenced plants are not late-flowering, elevated leaf number suggests accelerated leaf initiation rate in long days. VIGS treatments do not significantly affect leaf number under short-day conditions. *n* = 29–53 for *bars* and SDs. **d** Leaves are also initiated more quickly in *PhSBP1utr*-silenced plants relative to *PhSBP2utr*- and *PhCHS*-silenced individuals based on leaf counts at 22 and 33 days post-germination under long days. Increased leaf number at flowering correlates with late flowering in *PhSBP1utr*-silenced plants. *n* = 30–52 for *bars* and SDs. **e** Infection with *SBP1code* and *SBP1utr* VIGS vectors significantly reduces expression of *PhSBP1* (*red bars*) relative to *PhCHS* control plants (*dashed line*), but not *PhSBP2* (*orange bars*) or *PhCNR* (*purple bars*). *PhSBP2utr*-TRV2 infection causes gene-specific silencing of *PhSBP2*, whereas infection with the *PhSBP2code*-TRV2 vector causes silencing of *PhSBP2* and unexpected upregulation of *PhCNR*. *n* = 20 for *bars* and SDs. **f** *PhCHS*-silenced plant flowering at 68 days (*left*) versus *PhSBP1code*-silenced plant flowering at 151 days (*right*). **g** *PhCHS*-silenced (*left*) versus *PhSBP2code*-silenced (*right*) plant flowering at 75 days. Leaf node spacing is increased in *PhSBP1code* (**i**) and decreased in *PhSBP2code* (**j**) positive plants relative to *PhCHS*-silenced plants (**h**), consistent with leaf initiation rate. 33-day old *PhCHS* (**k**), *PhSBP1code* (**l**), and *PhSBP2code* (**m**) positive plants. *PhCHS*-silenced (**n**) flowering versus *PhSBP2utr*-silenced (**o**) late-flowering plant. *Errors* *bars* are standard deviations for multiple biological replicates. *Asterisks* denote significant differences at the *P* < 0.05 (*asterisk*), *P* < 0.01 (*double* *asterisk*), and *P* < 0.001 (*triple* *asterisk*) levels according to a Tukey’s (**e**) or Dunnett’s (**a**–**d**) test

To identify any functional differences following duplication in clade-VI *SPL* genes, virus-induced gene silencing (VIGS) was conducted in petunia targeting *PhSBP1, PhSBP2*, and the experimental control anthocyanin pathway gene *CHALCONE SYNTHASE* (*PhCHS*). Since the most efficient VIGS vectors have previously been found to match the coding region of target genes (Lu and Page 2008), we designed *PhSBP1* and *PhSBP2* silencing vectors that spanned the 3′-end of the coding regions (hereafter *PhSBP1code* and *PhSBP2code*). Additionally, to confirm specificity of gene silencing, we repeated experiments with *PhSBP1* and *PhSBP2* silencing vectors spanning the 3′-UTRs (hereafter *PhSBP1utr* and *PhSBP2utr*). Over 250 plants screened positive for petunia VIGS constructs, with an infection-efficiency of around 80 %. qRT-PCR analyses also revealed that infection was strongly negatively correlated with target gene expression (*P* < 0.001 for coding region vectors, *P* < 0.05 for 3′-UTR vectors, one-tailed Tukey’s test) (Fig. 3e) or, in the case of *PhCHS*-TRV2 infected control plants, with loss of petal anthocyanin production (Fig. 3g, n).

For plants infected with *SBP* constructs, levels of off-target *PhCNR* and *PhSBP1* (*PhSBP2code*-TRV2 and *PhSBP2utr*-TRV2) or *PhSBP2* (*PhSBP1code*-TRV2 and *PhSBP1utr*-TRV2) transcripts were not significantly decreased relative to *PhCHS*-*TRV2* infected plants (Fig. 3e). However, despite this evidence of no cross silencing among clade-VI *SPL* genes, *PhCNR* expression levels were significantly higher (*P* < 0.05, two-tailed Tukey’s test) in *PhSBP2code*- versus *PhCHS*-TRV2-infected plants (Fig. 3e). Based on these data, we infer that the *PhSBP2code*-TRV2 vector is less gene-specific than all the other VIGS vectors, resulting in the silencing of *PhSBP2* and an unknown negative regulator of *PhCNR*. We nonetheless continued phenotypic characterization of *PhSBP2code* silenced plants to gain possible insight into the effect of elevated *PhCNR* levels on plant development.

Consistent with our *SPL* transcript level screen, silencing of *PhSBP1* resulted in similar phenotypes for both the *PhSBP1code*- (Fig. 3a, c, f, i, l) and *PhSBP1utr*-TRV2 (Fig. 3b, d) vectors. *PhSBP1* silencing resulted in significantly delayed transition to inflorescence and flower development relative to *PhCHS* control plants based on a Dunnett’s test (Fig. 3a–d, f). Delays in phenology were observed in post-germination days to inflorescence and flower development (Fig. 3a, b), as well as in increased numbers of leaves and branches during flower emergence of *PhSBP1code*- and *PhSBP1utr*-TRV2-infected plants (Fig. 3c, d; Supplemental Fig. S3a). Although leaves became narrower over time in early developing wild type petunias (Supplemental Fig. S3b), possibly reflecting the transition from juvenile to adult growth as in Arabidopsis (Poethig 2003; Wu and Poethig 2006), there was no difference in leaf shape or trichome density between developmentally comparable leaves of *PhCHS*-TRV2 and *PhSBP1code*-TRV2-infected plants (Supplemental Fig. S3c). However, while *PhSBP1*-silenced plants had increased leaf and branch numbers at the onset of flowering due to the significant delay in flowering time (Fig. 3c, d; Supplemental Fig. S3a), analysis of pre-flowering leaf number demonstrated that *PhSBP1*-silenced plants had a reduced rate of leaf initiation. Specifically, at 22 and 33 days post-germination, *PhSBP1*-silenced plants had significantly fewer leaves than *PhCHS*-silenced plants, suggesting that leaf initiation was suppressed (Fig. 3c, d).

Plants positive for the *PhSBP2utr* vector flowered significantly later than *PhCHS*-silenced plants under long days, despite not being delayed in the transition to inflorescence development (Fig. 3b, n, o). However, in contrast to *PhSBP1*, silencing of *PhSBP2* using the *PhSBP2utr*-VIGS vector had no effect on the rate of leaf initiation under long days, at least during the initial 33 days post-germination (Fig. 3d). In plants infected with the *PhSBP2code*-TRV2 vector, where *PhSBP2* is silenced and *PhCNR* expression is enhanced (Fig. 3e), there was no difference in flowering time relative to control plants, but leaf number and branch number at flowering were significantly increased (Fig. 3a, c, g; Supplemental Fig. S3a). Thus, because down-regulation of *PhSBP2* alone (*PhSBP2utr* vector) leads to delayed flowering and no change in leaf or branch number, and down-regulation of *PhSBP2* with increased *PhCNR* (*PhSBP2code* vector) has no effect on flowering and increases leaf number, these results suggest that *PhCNR* functions similarly to *PhSBP1*, accelerating flowering and leaf initiation rate in wild type petunia.

To determine if photoperiod has an effect on VIGS phenotypes, we conducted a subset of similar experiments using the *PhCHS*-, *PhSBP1code*-, and *PhSBP2code* vectors under 8 h short-day conditions. With this reduced photoperiod, *PhCHS*-, *PhSBP1code*-, and *PhSBP2code*-silenced plants failed to flower completely (Fig. 3a). However, addition of gibberellic acid to 6-month-old short-day grown plants promoted flowering, with no evidence of leaf number differences at flowering time between plants positive for either of the constructs compared to control plants (Fig. 3c).

### Downstream targets of petunia clade-VI *SPL* genes are largely conserved

**Fig. 4:**
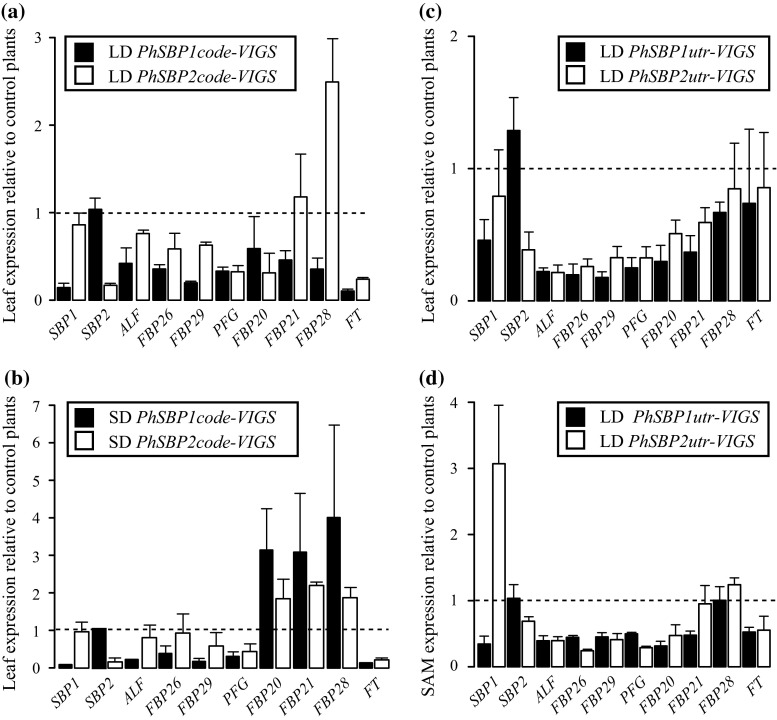
Effect of petunia clade-VI *SPL* gene silencing on putative target genes in leaves (**a**–**c**) and shoot apical meristems (SAM) (**d**) relative to *PhCHS*-silenced plants (*dashed lines*). *LD* plants grown under 16 h long days, *SD* plants grown under 8 h short days. *n* = 3 for *bars* and SDs

Differential regulation of downstream targets likely explains functional differences in phase change regulation and rate of leaf initiation between *PhSBP1* and *PhSBP2*, and the phenotypic differences between *PhSBP2utr*- and *PhSBP2code*-TRV2 infected plants. In order to determine what genes might be differentially affected, we examined changes in expression of known target orthologs (Klein et al. 1996; Wang et al. 2009; Yamaguchi et al. 2009; Preston and Hileman 2010) in response to *PhSBP1* and *PhSBP2* silencing, and/or increased *PhCHS* expression (Fig. 4). The youngest (upper) leaves of plants from different treatments were matched by leaf number (Fig. 4a–c) and transitional shoot apical meristems were matched by developmental stage (Fig. 4d).

In all *PhSBP1*- and *PhSBP2*-silenced plants grown under long days, the flower development genes *ALF, FBP26, FBP29, PFG,* and *FBP20* showed at least a mean 1.5-fold reduction in leaf and shoot apex expression relative to control plants (Fig. 4a, c, d). *FT* was also reduced at least 1.5-fold in long-day leaves of plants silenced with *PhSBP1code* and *PhSBP2code* constructs (Fig. 4a), and shoot apices of plants silenced with *PhSBP1utr* and *PhSBP2utr* constructs (Fig. 4d). However, silencing of either *PhSBP1* or *PhSBP2* with a 3′-UTR construct had little effect on *FT* expression in long-day grown leaves (Fig. 4c). Although expression of the *SOC1*-like genes, *FBP21* and *FBP28*, was consistently reduced in leaves of *PhSBP1*-silenced plants, expression levels relative to control plants varied between leaves infected with *PhSBP2utr* and *PhSBP2code* (Fig. 4a versus 4c) vectors. Whereas *PhSBP2utr*-VIGS plants had reduced *FBP21* and control-like *FBP28* expression levels, *PhSBP2code*-VIGS plants had control-like *FBP21* and elevated *FBP28* expression levels (Fig. 4a versus c). Under short days, infection with *PhSBP1code* and *PhSBP2code* vectors caused at least 1.5-fold silencing of leaf *FT*, *FBP29*, and *PFG*; *ALF* and *FBP26* only showed down-regulation when *PhSBP1* was silenced, and *FBP20* transcripts increased at least 1.5-fold in both *PhSBP1*- and *PhSBP2*-silenced plants (Fig. 4b). Taken together, these data suggest similar transcriptional targets for *PhSBP1* and *PhSBP2*, and support the idea that the wild type flowering time of *PhSBP2code*-TRV2 plants is due to the coincident downregulation of *FT, ALF,* and *AP1/FUL*-like genes, and upregulation of a *SOC1*-like gene.

## Discussion

Our study demonstrates that two petunia clade-VI *SPL* genes, *PhSBP1* and *PhSBP2*, have evolved in function following their duplication at the base of core eudicots. The effects of gene silencing on development are consistent with *PhSBP1* promoting inflorescence development, flower emergence, and leaf initiation in wild type petunias. In contrast, *PhSBP2* has no obvious effect on the timing of inflorescence development or leaf development, but positively affects the onset of flower production. Although not a direct test of *PhCNR* function, comparison of plants infected with the *PhSBP2utr* and *PhSBP2code* vectors, the latter of which increases *PhCNR* levels, suggests that *PhCNR* in wild type plants functions similarly to *PhSBP1* by promoting flowering and accelerating leaf initiation through decreased internode spacing. Unlike orthologs in Arabidopsis and *M. guttatus*, *PhSBP1* and *PhSBP2* transcript levels are unaffected by exogenous gibberellic acid treatment. However, increasing day length and developmental age positively regulates both genes, resulting in the transcriptional activation of a conserved set of target genes, including members of the *FUL, SOC1, LEAFY*, and *FT* clades of flowering time transcription factors (Klein et al. 1996; Corbesier and Coupland 2006; Corbesier et al. 2007; Wang et al. 2009; Yamaguchi et al. 2009; Preston and Hileman 2010; Preston et al. 2014).

Despite its efficacy in petunia, one important caveat to consider before making comparisons of clade-VI *SPL* gene function across species is that VIGS results in incomplete silencing that can vary spatially and temporally between plants. Thus, the silencing phenotypes described for *PhSBP1* and *PhSBP2* potentially underestimate the effects of these genes on development. Regardless of these limitations, we believe that the data presented here are meaningful in the sense that they can still reveal novel gene functions and, since several hundred plants were screened, distinguish the direction of developmental effects even for quantitative phenotypes. Similar levels of silencing between plants for *PhSBP1* and *PhSBP2* VIGS constructs also allow direct comparisons of petunia gene function following gene duplication. Ultimately, future studies exploiting powerful resources such as transposon-tagged petunia mutants or CRISPR-Cas site directed mutagenesis (Bortesi and Fischer 2015), will required to determine the overall effectiveness of VIGS approaches in petunia. These resources were unavailable to us at the start of this project.

Caveats notwithstanding, results of our study demonstrate a novel function for clade-VI genes in accelerating leaf initiation rate and a conserved function for these genes in promoting flower development. Specifically, whereas the Arabidopsis *SBP1*-like genes *AtSPL3, AtSPL4*, and *AtSPL5* are likely functionally redundant in promoting vegetative phase change and flowering, petunia *PhSBP1*, *PhSBP2*, *PhCNR* and snapdragon *AmSBP1* promote flowering, *PhSBP1/2* and *AmSBP1* control branching, *PhSBP1* and *PhCNR* promote leaf initiation, *PhSBP1* positively regulates late vegetative phase change, and tomato *CNR* promotes fruit ripening (Manning et al. 2006; Wu and Poethig 2006; Wang et al. 2009; Yamaguchi et al. 2009; Preston and Hileman 2010; this study). With the exception of fruit ripening and acceleration of leaf initiation, all of these functions have been described in analyses of more distantly related *SPL* homologs in Arabidopsis and rice (Schwarz et al. 2008; Shikata et al. 2009; Usami et al. 2009; Jiao et al. 2010; Miura et al. 2010). Thus, this evolutionary pattern is consistent with evolution mainly through differential sub-functionalization (Preston and Hileman 2013). Our data also demonstrate that clade-VI *SPL* genes differ in their response to the gibberellic acid signaling pathway, the lack of a response being correlated with perenniality. We suggest that future work focuses on the elucidation of downstream factors involved in functional diversification of clade-VI *SPL* genes following both speciation and duplication.

### *Author contribution statement*

J.C.P., S.A.J., and R.O. conducted the experiments; J.C.P and S.A.J. analyzed the data; and J.C.P. and L.C.H. conceived of/designed the experiments and wrote the article. All authors read and approved the manuscript.

## Electronic supplementary material

Supplementary **Supplemental Fig. S1** Expression of petunia clade-VI *SPL* genes and putative targets in floral organs and leaves. Black, white, and gray bars denote *SPL,* *ALF*, and *AP1/FUL*-like gene expression, respectively. *n* = 3 for bars and SDs (EPS 341 kb)

**Supplemental Fig. S2** Expression of clade-VI *SPL* genes from annual *M. guttatus* (**a, b**) and perennial snapdragon (**c, d**) in response to gibberellic acid (GA) treatment (white bars) under 8 h short days (SD). Only *MgSBP1* increases in expression in response to GA. *n* = 4–5 for bars and SDs (EPS 450 kb)

**Supplemental Fig. S3** Effect of gene silencing and developmental time on branch number and leaf shape. **a** Boxplot showing significant differences in branch number at flowering between *PhCHS*-, *PhSBP1code*-, and *PhSBP2code*-VIGS infected plants. **P < 0.01; ***P < 0.001 according to a one-tailed Dunnett’s test. **b** Mean leaf shape (ratio of laminar length to width) in a series of leaves from wild type petunia plants grown under long days (black bars) and short days with gibberellic acid (GA) addition (gray bars). Stars denote flowering plants. A one-tailed Dunnett’s test comparing long-day leaf shapes was significant (*P* < 0.05) between leaf numbers one and 12 to 64, denoted *a*. **c** Mean leaf shape in a series of leaves from *PhCHS*-TRV2- (black bars), *PhSBP1code*-TRV2- (gray bars), and *PhSBP2code*-TRV2-infected (white bars) plants. No significant differences were found across treatments, suggesting that clade-VI *SPL* genes do not affect leaf shape as a proxy for juvenile-adult growth. *n* = 6–10 for bars and SDs (EPS 375 kb)

Supplementary material 4 (DOC 36 kb)

Supplementary material 5 (DOC 40 kb)

Supplementary material 6 (DOCX 123 kb)

## Abbreviations

*ALF*: *ABERRANT LEAF AND FLOWER*
*CHS*: *CHALCONE SYNTHASE*
*FBP*: *FLORAL BINDING PROTEIN*
*FUL*: *FRUITFULL*
*FT*: *FLOWERING LOCUS T*
*SOC1*: *SUPPRESSOR OF OVEREXPRESSION OF CONSTANS 1*
*SBP*: *SQUAMOSA PROMOTER BINDING PROTEIN*
*SPL*: *SBP*-like
TRV: Tobacco rattle virus
VIGS: Virus-induced gene silencing
