# Supplementary material for: Paralogous SQUAMOSA PROMOTER BINDING PROTEIN-LIKE (SPL) genes differentially regulate leaf initiation and reproductive phase change in petunia
Source: Planta. 2015 Oct 7;243:429–40. doi: 10.1007/s00425-015-2413-2 (PMC4722060; doi:10.1007/s00425-015-2413-2)
Supplement: Supplementary file 4 — Supplementary material 4 (DOC 36 kb) [file 425_2015_2413_MOESM4_ESM.doc]

**Table S1** Primers used to amplify asterid clade-VI *SPL* and petunia *SQUA* genes

| **Primer name** | **Sequence (5’-3’)** | **Combination** | **Product** |
| --- | --- | --- | --- |
| *Forward:*  PhSBP2.deg.F  PhSBP1.deg.F  PotSBP2B.F  PotSBP1E.F    PhCNR.A.F  PetHySPL2.F  PetHySPL1.F  PhSBP1front1F  PhSBP1front2F  SBP2.166.F    SQUAdeg.F  PhSQUAdeg.F  PhSQUA.def.F  PhSBP2cd.Bam.F  PhSBP1cd.Bam.F  PhSBP1utr.Bam.F  PhSBP2utr.Bam.F  *Reverse:*  PolyTQT  PotSBP2D.R  PetHySPL2.R  PetHySPL1.R  SBP2.324.R  PhCNR.B.R  PhSQUAdeg.R  PhSQUA.def.R  PhSBP2cd.Xho.R  PhSBP1cd.Xho.R  PhSBP1utr.Xho.F  PhSBP2utr.Xho.F | CAGCGTTCTTGYCAGGTSGA  TAYTACCARAGGCACARGGT  TTGTSAAGTTCATGCTAAGGCT  TACCATCGCCGCCAYAARGT  AGCGATTCTGTCAGCAATGT  TGTTCTTGTTGCTGGCCTAA  CGCCACAAGGTCTGTGAAT  GAAGAGGATARCAAAAGRAGGGT  TCCCTCTGGKAGGAAGCTA  GTYTGTGARKTTCATGCCAAG  TGYGATGCTGAAGTTGCTTT  ATTGTYTTCTCTCANAAGGG  TGAAGAGGATAGAGAACAAG  ACAGGATCCCAGCAATGTAGCAGGTTCCA  ACAGGATCCCTTGGCAGGCCATAATGAAC  ATAGGATCCCCTGGAGAAGGATCAATTTAAGG  ATAGGATCCTTCTGTCATTTCCTACCTTCGTT  GACTCGACTCGACATCGAT^17^V  TTGCGACGCCGCTTGTTGTGTCC  TAAGCTTTGGCGTCCCTTTA  GTTCATTATGGCCTGCCAAG  CTTNCGNCGCCGCTCATTGTG  AGAGAGCTTGCTTAAACCACA  CCTTCTCYTTRATCTTCTT  CAWGCRGCRAAGCAKCCAAG  CTCCTCGAGGAGAGTAAGCATTCAGAAGGACA  CTCCTCGAGTTTTGGTTAAAGGGAAATTTGG  AGTCTCGAGTTCCATTCAAGTGAAATGAAATTA  AGTCTCGAGGCATGCAAGAAAGTTCCATT | PolyTQT  PolyTQT  PotSBP2D.R  PotSBP2D.R  PhCNR.B.R  PetHySPL2.R  PetHySPL1.R  PolyTQT  PolyTQT  SBP2.324.R  PolyTQT/PhSQUAdeg.R  PolyTQT/PhSQUAdeg.R  PhSQUA.def.R  PhSBP2.Xho.R  PhSBP1.Xho.R  PhSBP1utr.Xho.F  PhSBP2utr.Xho.F  See above  See above  See above  See above  See above  See above  See above  See above  See above  See above  See above  See above | *PhSBP2*  *PhSBP1*  *PhSBP1, PbSBP1, RtSBP1, DicSBP1*  *PhSBP2, DcSBP1, PbSBP1, RtSBP1, BtSBP1, DicSBP1, PrhSBP1, PfSBP1*  *PhCNR*  *PhSBP2*  *PhSBP1*  *PhSBP1*  *PhSBP1*  *PhSBP2, RtSBP2, DcSBP2, DcSBP3, PbSBP3*  None  None  None  *PhSBP2code* for VIGS  *PhSBP1code* for VIGS  *PhSBP1utr* for VIGS  *PhSBP2code* for VIGS |
