# Supplementary material for: Paralogous SQUAMOSA PROMOTER BINDING PROTEIN-LIKE (SPL) genes differentially regulate leaf initiation and reproductive phase change in petunia
Source: Planta. 2015 Oct 7;243:429–40. doi: 10.1007/s00425-015-2413-2 (PMC4722060; doi:10.1007/s00425-015-2413-2)
Supplement: Supplementary file 5 — Supplementary material 5 (DOC 40 kb) [file 425_2015_2413_MOESM5_ESM.doc]

**Table S2** Primers for qRT-PCR. All primers were tested for PCR efficiency; underlined primers with high efficiency were used for quantification. Bold primers are based on the Genbank accession GU939627.1.

| **Primer name** | **Sequence (5’-3’)** | **Combination** | **Product** |
| --- | --- | --- | --- |
| *Forward:*  PhEF1a.F  PhSBP2.rt.F  PhSBP1.rt12.F  PhCNRq.A.F  PhPFG.672.F  PhFUL.29.F  PhFL.40.F  PhFL.27.F  PhALF.1056.F  PhFBP26.29.F  PhFBP29.135.F  **PhFTqRT.F**  PhFBP20.F  PhFBP21.F  PhFBP28.F  *Reverse:*  PhEF1a.R  PhSBP2.404.R  PhSBP1.rt.176R  PhCNRq.A.F  PhPFG.891.R  PhFUL.195.R  PhFL.205.R  PhFL.203.R  PhALF.1215.R  PhFBP26.195.R  PhFBP29.342.R  **PhFTqRT.R**  PhFBP20.R  PhFBP21.R  PhFBP28.R | See Snowden et al. (2005)  AAAGGGACGCCAAAGCTTAC  CAGAACAAGGGTCTTGACTCG  AGCGATTCTGTCAGCAATGT  CACAGCCATTGGACTCTCCT  AGCTAGCCCAGCAGAGTCAG  CGTCCACAACTCGAACAACA  TGCTGAGGTTGCTTTGATTG  GGTAGCCATAGCTGCTCGAC  AGCTAGCCCAGCAGAGTCAG  GGCTTTTGGAGCAAAAACAG  CAAGGCCTACCATGGGAATA  GCTCAAGTTGGATTGGTCATT  ATCCTTGCTGCTGAAAATGC  GACGCAGCAAGTCTGATGAA  See Snowden et al. (2005)  TGCATGCAAGAAAGTTCCATT  AGTACTGCCGGAGACTTTGC  TGTGGATGCTGCTTCAACTT  AGCTTCGAACCCGTGATATG  CACAGTGTTTGGTGGTTGCT  AAGGTGGCATGAGTGGATTG  ATGAGCTTGGGGTGTTCAAG  ACCACCACCAGAAACTGAGG  CACAGTGTTTGGTGGTTGCT  TGAGACACAATGGGTTCCAA  CATGATACGACGACCACCAG  CAAACCTGCTGTCTCATGCT  CTCAAGCTATGCATCCAACG  CCACAGCATTGCATTTTCAG | PhEF1a.R  PhSBP2.404.R  PhSBP1.rt.176R  PhCNRq.A.R  PhPFG.891.R  PhFUL.195.R  PhFL.205.R  PhFL.203.R  PhALF.1215.R  PhFBP26.195.R  PhFBP29.342.R  PhFTqRT.R  PhFBP20.R  PhFBP21.R  PhFBP28.R  See above  See above  See above  See above  See above  See above  See above  See above  See above  See above  See above  See above  See above  See above  See above | *PhEF1α*  *PhSBP2*  *PhSBP1*  *PhCNR*  *PhPFG*  None  None  None  *PhALF*  *PhFBP26*  *PhFBP29*  *PhFT*  *PhUNS*  *PhFBP21*  *PhFBP28*  *PhEF1α*  *PhSBP2*  *PhSBP1*  *PhCNR*  *PhPFG*  None  None  None  *PhALF*  *PhFBP26*  *PhFBP29*  *PhFT*  *PhUNS*  *PhFBP21*  *PhFBP28* |
