# Supplementary material for: Paralogous SQUAMOSA PROMOTER BINDING PROTEIN-LIKE (SPL) genes differentially regulate leaf initiation and reproductive phase change in petunia
Source: Planta. 2015 Oct 7;243:429–40. doi: 10.1007/s00425-015-2413-2 (PMC4722060; doi:10.1007/s00425-015-2413-2)
Supplement: Supplementary file 6 — Supplementary material 6 (DOCX 123 kb) [file 425_2015_2413_MOESM6_ESM.docx]

**Table S3** Newly generated *SPL* sequences under 200 bp in length

| Species | Gene | Sequence (5'-3') |
| --- | --- | --- |
| *Bidens torta* | *BtSBP1* | TACCATCGCCGCCACAAGGTTTGCGAATTCCATGCCAAGGCTACGGTTGTTCTTCTTTCCGAGTTACGGCAGCGG  TTCTGTCAGCAGTGCAGCAGGTTCCATGAGTTATCAGAGTTCGACGAAGCTAAAAGAAGTTGCCGAAGGCGTTTG  GCGGGACACAACAAGCGGCGTCGCAAA |
| *Delosperma cooperi* | *DcSBPL* | TTCTGTCAGCAATGCAGCAGGTTCCATTTGCTGGGTGAATTTGATGACTGTAAGCGTAGCTGTCGTAAACGCCTT  GCCGGGCACAATGAGCGGCGCCGCAAG |
| *Delosperma cooperi* | *DcSBP2* | TTCTGCCAGCAATGCAGCAGGTTTCACGAGCTATCCGAATTCGACGAGACAAAGAGGAGTTGCCGGAGGCGTCTG  GCGGGCCACAATGAGCGGCGCCGTAAG |
| *Dianthus caryophyllus* | *DicSBP1* | TACCATCGCCGCCATAAGGTGTGTGAGTTCCATGCTAAGGCTCCCTCCGTGTTGGTCTGTGGGCTACGACAACGT  TTCTGCCAGCAATGTAGCAGGTTTCATGAACTGTCGGAGTTCGACGAGGCAAAGAGGAGCTGCAGGAGGCGGCTA  GCAGGACACAACAAGCGGCGTCGCAAA |
| *Penstemon barbatus* | *PbSBP4* | TTCTGTCAGCAATGCAGCAGGTTTCATCAATTGCCTGAATTTGACCAAGGAAAACGAAGTTGCCGCAGACGCCTT  GCTGGCCACAATGAGCGGCGGCGGAAG |
| *Penstemon barbatus* | *PbSBP1* | CGCTTTTGCCAGCAGTGCAGCAGGTTTCATGAGTTATCAGAGTTTGATGAAGCGAAGAGAAGTTGTCGTAGGCGT  TTGGCGGGACACAACAAGCGGCGTCGCAAAAGCCGTTGCTGTTCTGAAGGTAGAATCCTGCCATCT |
| *Plumeria rubra* | *PrSBP1* | TACCATCGCCGCCATAAGGTTTGCGAATTCCATGCCAAGGCTACGGTTGTTCTTCTTTCCGAGTTACGGCAGCGG  TTCTGTCAGCAGTGCAGCAGGTTCCATGAGTTATCGGAGTTCGACGAAGCTAAAAGAAGTTGCCGAAGGCGTTTG  GCGGGACACAACAAGCGGCGTCGCAAA |
| *Primula hortense* | *PrhSBP1* | TACCATCGCCGCCACAAGGTGTGCGAATTCCATGCCAAGGCTCCGGTCGTGATTGTCGCCGGAAATTGCCAGCGC  TTTTGCCAGCAATGCAGCAGGTTCCATGAGCTGTCAGAATTCGACGATACAAAGAGGAGTTGTCGAAGGCGGCTG  GCTGGACACAACAAGCGGCGTCGCAAA |
| *Ruellia trittoniana* | *RtSBP2* | TTCTGTCAGCAATGCAGCAGGTTTCACGAGATCTCCGAATTCGACCAATCCAAGAGAAGTTGCCGCAGGCGATTG  GCCGGCCACAATGAGCGGCGGCGGAAG |
